# Utilization of Assisted Reproductive Technologies in Breeding Auliekol Cattle: A Comparative Study

**DOI:** 10.3390/life14091167

**Published:** 2024-09-15

**Authors:** Altyn Kulpiisova, Kairly Yessengaliyev, Gulsara Kassimova, Ainat Kozhakhmetova, Bakytkanym Kadraliyeva, Abeldinov Rustem, Alma Temirzhanova, Nadezhda Burambayeva, Salbak Chylbak-ool, Elena Pakhomova, Nurzhan Abekeshev, Gulnara Baikadamova, Zhomart Kemeshev, Alexandra Tegza, Arman Issimov, Peter White

**Affiliations:** 1 Department of Veterinary Medicine, A. Baitursynov Kostanay Regional University, Kostanay 110000, Kazakhstan; altyn977@mail.ru; 2 Department of Veterinary Medicine, Zhangir Khan West Kazakhstan Agrarian Technical University, Oral 090000, Kazakhstan; 3 Department of Zootechnology, Genetics and Breeding, Toraighyrov University, Pavlodar 140000, Kazakhstan; 4 Department of Veterinary Medicine, Russian State Agrarian University-Moscow Timiryazev Agricultural Academy, Moscow 127343, Russia; 5 Department of Veterinary Medicine, Saken Seifullin Kazakh Agrotechnical University, Astana 010000, Kazakhstan; 6 Department of Biology, K.Zhubanov Aktobe Regional University, Aktobe 030000, Kazakhstan; 7 Sydney School of Veterinary Science, Faculty of Science, University of Sydney, Sydney 2006, Australia

**Keywords:** IVEP, embryo, IVF, Auliekol cattle

## Abstract

This study evaluates the utilization of in vitro embryo production (IVEP) technology for the conservation and breeding of the Auliekol cattle breed, a primary beef breed in Kazakhstan facing population decline due to the cessation of breeding programs and the incursion of transboundary diseases. We assessed the effect of consecutive ovum pick-up (OPU) procedures on oocyte yield and embryo production in Auliekol and Aberdeen Angus cows. A total of 2232 and 3659 oocytes were aspirated from Auliekol and Aberdeen Angus donors, respectively, with significantly higher yields and embryo production observed in Aberdeen Angus cows. The application of a meiotic block using Butyrolactone I (BLI) and subsequent in vitro fertilization (IVF) protocols was employed, with embryo development monitored up to the morula/blastocyst stage. Results indicated that Auliekol cows exhibited lower oocyte recovery, cleavage, and blastocyst rates compared to Aberdeen Angus cows, likely due to genetic characteristics. Despite the challenges, IVEP presents a valuable tool for the preservation and future propagation of the Auliekol breed, highlighting the need for further research to enhance reproductive outcomes and conservation strategies.

## 1. Introduction

Assisted reproductive technologies (ARTs) for livestock and wildlife species have been proposed as a strategy to address the challenges associated with maintaining the populations of endangered species [1]. In vitro embryo production significantly enhances the reproductive efficiency of economically valuable livestock [2]. In Kazakhstan, the population of the Auliekol breed has significantly declined over the past three decades. This decrease in the productivity of the entire livestock sector has been primarily attributed to the cessation of breeding programs and the emergence of transboundary diseases such as lumpy skin disease and foot and mouth disease during the post-Soviet period [3,4,5]. The Auliekol cattle is one of the primary beef breeds in Kazakhstan. The breed possesses valuable characteristics such as high growth potential, disease resistance, thermal adaptation, unassisted calving, and adaptability to local conditions. The breeding program started in the 1960s, and the desired breed was obtained in the 1990s by crossing Kazakh Whiteheaded cows with Charolais and Aberdeen Angus bulls [6]. 

In conservation biology, breeding live animals under IVEP management holds significant potential in the preservation of endangered wild or domestic species and breeds [1,7]. The main limitations of this conservation method, however, are inbreeding and the genetic drift associated with small population sizes, which lead to reduced embryo production and increased embryonic mortality in threatened species [8,9,10]. Moreover, by utilizing recent advancements in conservation biology, semen from desired bulls can be preserved through vitrification techniques [11]. Although semen preservation offers economic and quality advantages, it presents limitations in herd regeneration, requiring extensive time for the evaluation of another breed over multiple generations through backcrossing [12]. Accordingly, the preservation of threatened breeds via embryo conservation shows promise, requiring a single generation for breeding recovery.

The aim of this study, therefore, was to apply IVEP technology to evaluate its suitability for the conservation and continued breeding of the Auliekol breed. The effect of consecutive ovum pick-up (OPU) procedures per donor animal on oocyte yield and embryo production was also assessed. 

## 2. Materials and Methods

### 2.1. Animal Ethics

The protocol was approved by the Committee on the Ethics of Animal Experiments of the Marat Ospanov West Kazakhstan State Medical University (permit number: 05-07-05-20-2023).

### 2.2. Experimental Animals

In the present study, healthy, non-pregnant, cycling Auliekol (*n* = 70) and Hereford (*n* = 70) cows, approximately 48–54 months old and with high genetic merit, were used. Both cattle breeds were maintained in the same conditions on fenced pastures and access to water and concentrates were ad libitum. Before the experiment, animals underwent thorough examinations for reproductive abnormalities through ultrasonography and rectal palpation. No hormonal stimulants were used during OPU sessions in donor animals.

Ovum pick up sessions were carried out according to Seisenov, Duimbayev [13]. In brief, the perineal area was sterilized with 70% ethanol, and 3 mL of 2% lidocaine was injected into epidural space to reduce muscle contractions. To mitigate the thermal stress effects of the ambient environment on COCs, meteorological data were monitored using http://www.pogodaiklimat.ru/monitor.php (accessed on 20 June 2023). A summary of the meteorological data for the area is included as a Supplementary File.

### 2.3. Follicular Aspiration

Ovum pickup procedure (OPU) was conducted following the protocol published by Seneda, Zangirolamo [14]. Five OPU sessions were carried out on each donor animal at intervals of 15 days. The cattle were restrained, and antral follicle count (AFC) was carried out by a single technician using a real-time Aloka SSDV 500 ultrasonographic scanner with a 7 MHz transducer and a stainless steel guide. Oocytes were aspirated from visible follicles by puncturing them with a disposable 17-gauge needle (Hamilton, NV, USA) connected to a 5 cm diameter filter disc and a 100 mL conical tube (Sigma-Aldrich Inc., Maryland, MD, USA) and washed out by a vacuum set at 11–13 mL of water/min. The follicular contents aspirated were then transferred to the laboratory according to the method recommended by Guemra, da Silva Santo [15]. 

### 2.4. Meiosis Block with Butyrolactone I (BLI)

To evaluate the effect of BLI on COC meiosis inhibition, oocytes from each donor breed were grouped into four pools, each containing 30 COCs. These were designated as control groups and were transported from the field to the lab without BLI medium. Conversely, all other oocytes from each donor subjected to a meiotic block were cultured in vitro using BLI (B Sigma-Aldrich Inc., MD, USA) at a concentration of 25 µL in TCM199 medium (Sigma-Aldrich Inc., MD, USA), supplemented with 0.2 mM pyruvate. In the laboratory, oocytes from each donor breed were also grouped into four pools, each containing approximately 30–35 COCs. These pools were randomly selected to create meiosis block groups for further comparison of oocyte developmental stages with control groups.

### 2.5. In Vitro Embryo Production

Cumulus-oocyte complexes (COC) were examined for morphological features by an inverted stereomicroscope according to the protocol published by Seneda, Esper [16]. Oocytes that demonstrated one or two cumulus cell layers were selected for further IVF manipulations, whereas those displaying atretic or degradation characteristics were excluded.

Oocytes categorized for IVF (*n* = 500) were then washed in a 10% fetal calf serum-supplemented TCM-199-HEPES maturation medium (Sigma-Aldrich Inc., MD, USA) and antibiotics, including 50 μg/mL gentamicin, 22 mg/mL sodium pyruvate, and 83.4 mg/mL amikacin. Pools of 50 washed oocytes were then incubated for 24 h in 100 µL drops of an in vitro maturation (IVM) medium overlaid with mineral oil (Thermo Fisher Scientific, Waltham, MA, USA) under 5% CO_2_. The experiment was conducted in triplicate.

After IVM, the COCs were washed in PBS and introduced into a Tyrode lactate pyruvate (TALP) solution (Thermo Fisher Scientific, Waltham, MA, USA) in the presence of 0.5 µM penicillamine, 0.25 µM hypotaurine, 25 μM epinephrine, 83.4 mg/mL amikacin, and 22 mg/ mL sodium pyruvate. 

For the IVF procedure, Auliekol sires (*n* = 3) were used as a source of fresh semen. The obtained semen was purified using GM501 Gradient 45% and 90% (IVF Store, Alpharetta, GA, USA). The purified semen was then diluted in Tyrode medium supplemented with 10 mg/mL heparin and centrifuged for 30 min at 300 rpm. Next, sperm motility of no less than 95% and a concentration level of 2 × 10^7^ live spermatozoa per mL were adjusted using AndroVision (Minitube, Smythesdale, Australia) for further IVF. Fertilization was performed in groups of up to 30 matured oocytes in 150 µL microdrops.

After the IVF procedure, presumptive zygotes underwent cumulus cell removal by rinsing with a TCM-199 HEPES medium. Pools of 10–15 presumptive zygotes were then placed into 100 µL microdrops of an SOF-BE2 (Sigma-Aldrich Inc., MD, USA) fluid, covered with mineral oil, and subsequently incubated at 39 °C in an atmosphere containing 5% CO_2_. Presumptive zygotes were regularly monitored for embryonic development. The classification of the embryos’ developmental stages was conducted according to the protocol described by Wright [17].

### 2.6. Embryo Transfer (ET) and Pregnancy Diagnosis

**Figure 1:**
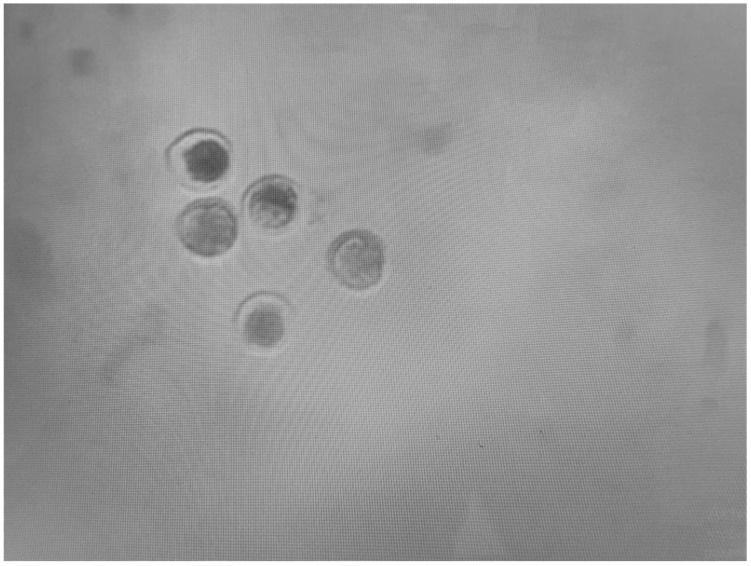
Inverted microscopic picture of Blastocysts produced in vitro on the seventh day of development. DIC magnification ×100.

Twenty Kazakh Whiteheaded cows and thirteen Aberdeen Angus cows were used as recipients for ET manipulation. The experiment was conducted in two replicates, resulting in a total of 40 transfers in Kazakh Whiteheaded cows and 26 transfers in Aberdeen Angus cows. In total, surrogate animals received 132 embryos that had developed to the late morula/blastocyst stage (Figure 1). The embryo transfer protocol described by Bárbara Letícia Marchi da Silva [18] was applied to prepare the recipient animals. To do so, the cows were administered an estradiol benzoate (3 mg) and a CIDR progesterone device (Pfizer, La Jolla, CA, USA) on day 0. On day 7, immediately following the removal of the progesterone implant, the cows were administered 300 IU of equine chorionic gonadotropin (eCG), 150 µg of d-cloprostenol, and 1 mg of estradiol cypionate (Pfizer, CA, USA). On day 18 following the initiation of the estrus synchronization protocol, each recipient cow received two fresh embryos, which were vaginally transplanted into the uterine horn on the same side as the corpus luteum. Recipient animals with a corpus luteum measuring 10 cm or greater in diameter were selected for embryo transfer. 

The initial pregnancy assessment was conducted via ultrasonography on days 27–30 following embryo transfer. On days 60 and 90 following embryo transfer, animals that tested positive for pregnancy in their initial screening underwent subsequent ultrasonographic evaluations to confirm gestation. 

### 2.7. Statistics

Graphs were compiled and statistical analyses were conducted using Prism 9 software (GraphPad Software, Inc., San Diego, CA, USA). The data underwent evaluation through a variety of statistical tests, including Student’s *t*-test and 2-way ANOVA with Tukey’s post hoc analysis. The quantity of oocytes and embryos obtained from donors subjected to OPU are presented as the mean ± SEM here. Statistical significance between groups is denoted using asterisks when *p* < 0.05, as detailed in the respective figures. 

## 3. Results

The results obtained from IVEP are presented in Table 1. A total of 2232 oocytes were aspirated from Auliekol donors, and 3659 oocytes were collected from Aberdeen Angus donors. Each cow was subjected to five OPU sessions at intervals of 15 days.

In the Auliekol cows, the number of oocytes obtained per OPU session ranged from 1 to 17. Oocytes categorized as viable were allowed to mature and then fertilized. On day 4 following fertilization, 899 embryos had cleaved, accounting for 62% of the total fertilized oocytes. Of the cleaved embryos, 189 (13%) progressed to the late morula/blastocyst stage by day 7 following fertilization, with an average yield of 2.7 (3.8%) embryos per donor cow in five OPU sessions. Ultrasonographic examination on day 30 following fertilization revealed that seventeen recipient cows were pregnant. By day 90, pregnancy was confirmed in four cows. Two calves were delivered at the end of the gestation period, while two other cows experienced a late second-trimester abortion. In Aberdeen Angus cows, ultrasonographic examination on day 30 following fertilization indicated pregnancy in eleven recipients. Of these, seven cows successfully carried their pregnancies to term and delivered live calves.

Over a total of five OPU sessions, variables such as the number of COCs recovered, viable oocytes, cleaved oocytes, and the morula/blastocyst ratio were significantly greater in Aberdeen Angus cows (*p* < 0.03) compared to Auliekol cows. Additionally, all variables were significantly greater in the Aberdeen Angus group (*p* < 0.001) compared to the Auliekol group when comparisons were made for each OPU session. Moreover, the mean ± SEM number of oocytes aspirated (10.6 ± 2.05 vs. 6.4 ± 1.12), viable oocytes (8.9 ± 1.42 vs. 4.1 ± 1.50), cleaved oocytes (6 ± 0.55 vs. 2.5 ± 0.35), and embryos developed into the morula/blastocyst (1.2 ± 0.22 vs. 0.5 ± 0.12) per variable was significantly higher (*p* ≤ 0.004) in the Aberdeen Angus group compared to the Auliekol group.

Regarding BLI treatment, embryo yields in Auliekol and Aberdeen Angus cattle showed no significant difference within each breed (*p* = 0.776) in morula/blastocyst rates on the seventh day of culture, regardless of the treatment applied (Table 2).

## 4. Discussion

In vitro embryo production has emerged as the most rapidly advancing technique in recent years, offering broad-scale applications for preserving endangered domestic and wild species and breeds [19,20]. To the best of our knowledge, this study represents the first utilization of IVEP technology in Auliekol cattle. 

The implementation of ex situ conservation programs for spermatozoa, oocytes, and embryos offers a considerable advantage by enabling the resumption of their development at a desired time [21,22]. More recent study indicate that cryoconservation is often inefficient due to the elevated risk of damage to reproductive materials [23]. Consequently, our initial research primarily focused on embryo production and birth rates. Nevertheless, the significance of cryoconservation for Auliekol embryos remains pertinent and warrants evaluation in future studies.

Given the scarcity of available studies on Auliekol cows, further research is warranted to explore their reproductive potential to both in vitro and in vivo embryo transfer techniques. The Multiple Ovulation and Embryo Transfer protocol, as outlined by Pontes, Nonato-Junior [24], was initially employed for embryo production. However, due to a poor response to treatment, this method was not utilized for embryo collection. This observation highlights the need for adapting and optimizing superovulation protocols specifically for Auliekol cows to enhance their reproductive performance. 

Our study showed that Auliekol cows demonstrated a lower number of transferable embryos per animal (2.7) compared to the previous studies, with averages of 3.2, 4.7, and 11.4 embryos per animal, respectively [25,26,27]. This discrepancy could be attributed to the increased oocyte yield observed in their studies due to the application of exogenous hormones. In the current study, a significant reduction in the number of oocytes aspirated was observed throughout OPU sessions in Auliekol cows. This observation aligns with findings reported by Monteiro, Batista [28]. Viana [29] noted that the reduction in the number of aspirated oocytes could be attributed to repeated ovarian punctures during consecutive OPU sessions. They hypothesized that repeated punctures in animals with extensive follicular development could lead to ovarian damage and reduce its function. 

In Auliekol cows, the mean number of oocytes recovered per OPU session (4.1) was significantly lower compared to the Aberdeen Angus breed (8.9) and those reported by Seisenov, Duimbayev [13], Pontes, Silva [30], and Viana [31]. Similarly, Auliekol cows showed a lower average percentage of viable oocytes (65%) compared to the Aberdeen Angus breed (76.6%). Conversely, the mean proportion of viable oocytes in Auliekol cows was comparable to those reported in other studies for Holstein (54%), Gir (70%), and Nellore (68%) breeds [30,32]. Moreover, the Auliekol cows demonstrated significantly lower cleavage and blastocyst rates compared to those in Holstein and Nellore cows [32,33]. Overall, our findings exhibited some similarities with the aforementioned studies. However, the Auliekol cows showed lower cleavage and blastocyst rates, leading to a reduced birth rate.

In the present study, the COC was transported using a maturation block, which could be detrimental to the gametes of Auliekol cattle. Therefore, the low blastocyst formation rate could be due to the meiosis blocking medium using BLI (12 µM BLI, TCM-199 without HEPES supplemented with 50 mg/mL gentamicin, 0.2 µM pyruvate, and 100 µM cysteamine). For this reason, the effect of a temporary induced meiosis block on oocytes obtained by OPU from Auliekol cattle was evaluated in this study. The percentage of viable oocytes that developed into blastocysts did not differ within breed between nonblocked (control) and blocked groups, which is similar to the findings reported by Guemra, da Silva Santo [15,34,35]. However, previous studies utilizing butyrolactone support the notion that this strategy would facilitate extended oocyte transport (e.g., from herds located far from the laboratory) and enable oocyte collection over two consecutive days for a single IVF and embryo transfer routine [15,36].

Our study demonstrated that Auliekol cattle exhibited lower cumulus-oocyte complex production rates, efficiency of IVEP, and morula/blastocyst development rates compared to Aberdeen Angus cattle. These findings could be attributed to the inherent genetic characteristics of the breed [37] and the hybrid vigor in embryos derived from Angus cows [13], as opposed to the purebred embryos of Auliekol cows. Another potential explanation could be the heat stress effect on female bovine [25]. The fact is that, on the farm, where donor animals are maintained, 24 h open air pasture was practiced. Animals had free access to barns that were equipped with climate control. In previous study, it was demonstrated that systemic heat stress exposure results in elevated body temperature that diminishes the follicular population and reduces the size of preovulatory follicles. Additionally, even a slight increase in follicular temperature within the ovaries can inhibit ovulation [38]. High temperatures also cause damage to cellular organelles and DNA within the oocyte [39], and during the early stages of embryonic development, they induce cellular and epigenetic alterations that restrict both embryo development and quality [40]. As previously noted, there are no references available regarding the application of IVEP in Auliekol cattle. We observe that the low pregnancy rate relative to the number of aspirated oocytes Auliekol breed is similar to that exhibited by Kazakh Whiteheaded breed [13]. This peculiarity could be explained by the fact that Kazakh Whiteheaded cattle were involved in the breeding program for the Auliekol breed [6], and their inferior reproductive performance could be genetically inherited. Despite the generally low and highly variable efficiency of reproductive biotechnologies, these methods are crucial for species recovery programs, ensuring long-term genetic and demographic sustainability, as well as securing food demand on a nationwide scale [2,41,42].

## Figures and Tables

**Table 1:** Summary of the IVEP experiment in Auliekol and Aberdeen Angus cows.

Variables	Auliekol Cows (*n* = 70), OPU (1–5)					Aberdeen Angus Cows (*n* = 70), OPU (1–5)				
	1	2	3	4	5	1	2	3	4	5
Oocytes aspirated/mean per animal	566/ 8.1 ± 2.2	480/ 6.8 ± 3.6	471/ 6.7 ± 4.3	381/ 5.4 ± 2.4	334/ 4.8 ± 1.1	842/ 12.0 ± 5.4 *	722/ 10.3 ± 4.1 *	774/ 11.1 ± 5.4 *	711/ 11.0 ± 3.7 *	610/ 8.7 ± 2.6 *
Viable oocytes/mean per animal	403/ 5.6 ± 1.5	302/ 4.3 ± 0.5	320/ 4.6 ± 2.2	221/ 3.2 ± 0.4	205/ 2.9 ± 0.6	689/ 9.9 ± 3.7 *	457/ 6.5 ± 2.7 *	583/ 12.3 ± 5.2 *	554/ 8.3 ± 1.3 *	520/ 7.4 ± 2.4 *
Cleaved oocytes/mean per animal	277/ 4.0 ± 0.2	201/ 2.9 ± 1.2	191/ 2.7 ± 1.3	108/ 1.5 ± 0.2	122/ 1.7 ± 0.7	472/ 6.7 ± 4.1 *	344/ 4.9 ± 2.4 *	443/ 6.3 ± 2.1 *	404/ 5.8 ± 2.2 *	419/ 6.0 ± 2.7 *
Morula/blastocyst on day 7	66/ 0.9 ± 0.3	54/ 0.8 ± 0.5	24/ 0.3 ± 0.2	33/ 0.5 ± 0.4	12/ 0.2 ± 0.1	112/ 1.6 ± 0.6 *	106/ 1.5 ± 1.0 *	76/ 1.1 ± 1.2 *	67/ 0.9 ± 0.4 *	71/ 1.01 ± 0.7 *

* 2-way ANOVA and Sedak multiple comparison tests.

**Table 2:** In vitro embryo production from oocytes retrieved via OPU from Auliekol and Aberdeen Angus donors, with or without BLI treatment prior to in vitro maturation.

Treatments	Number of Oocytes	Morula/Blastocyst (% + SD)
Control (Auliekol)	120	24 (20 ± 2.4)
Miosis Block (Auliekol)	130	29 (22.3 ± 4.4)
Control (Aberdeen Angus)	120	37 (31 ± 1.12)
Miosis Block (Aberdeen Angus)	130	41 (31.5 ± 2.56)

## Data Availability

The original data presented in the study are openly available in FigShare at 10.6084/m9.figshare.27020320.

## Supplementary Materials

The following supporting information can be downloaded at: https://www.mdpi.com/article/10.3390/life14091167/s1, Table S1: Summary of meteorological data on the area studied.
