# Supplementary material for: Utilization of Assisted Reproductive Technologies in Breeding Auliekol Cattle: A Comparative Study
Source: Life (Basel). 2024 Sep 15;14(9):1167. doi: 10.3390/life14091167 (PMC11433346; doi:10.3390/life14091167)
Supplement: Supplementary file 1 [file life-14-01167-s001.zip › life-3139498-supplementary.pdf]

Table S1. Summary of meteorological data on the area studied.

Months	Air temperature, °C				Precipitation, mm
	Minimum	Medium	Maximum	Deviation from the Norm	
June 2022	10	21.4	35.3	+2.3	3.0
July 2022	18.5	26.2	37.2	+1.1	0.0
August 2022	17.5	23.4	28.1	+0.2	5.0
September 2022	7.2	16.5	30.5	-0.3	7.0
